# A Real‐Time Cell Death Self‐Reporting Theranostic Agent for Dynamic Optimization of Photodynamic Therapy

**DOI:** 10.1002/advs.202417678

**Published:** 2025-03-08

**Authors:** Wei Bian, Qiyue Wang, Cui He, Pan Tao, Juanjuan Zheng, Yulu Zhang, Jing Li, Fangyuan Li, Hongyan Jia, Daishun Ling

**Affiliations:** ^1^ Department of Breast Surgery First Hospital of Shanxi Medical University Taiyuan 030001 China; ^2^ Key Laboratory of Cellular Physiology at Shanxi Medical University Ministry of Education Taiyuan 030000 China; ^3^ Frontiers Science Center for Transformative Molecules School of Chemistry and Chemical Engineering School of Biomedical Engineering National Center for Translational Medicine Shanghai Jiao Tong University Shanghai 200240 China; ^4^ Department of Clinical Laboratory Songjiang Research Institute Shanghai Key Laboratory of Emotions and Affective Disorders (LEAD) Songjiang Hospital Affiliated to Shanghai Jiao Tong University School of Medicine Shanghai 201600 China

**Keywords:** fluorescence imaging, imaging‐guided surgery, nanosensor, photodynamic therapy, theranostic agent

## Abstract

The therapeutic efficiency of photodynamic therapy (PDT) hinges on the drug‐light interval (DLI), yet conventional approaches relying on photosensitizer accumulation often lead to suboptimal irradiation and adverse side effects. Here, a real‐time cell death self‐reporting photodynamic theranostic nanoagent (CDPN) is presented that dynamically monitors extracellular potassium ion ([K⁺]_ex_) fluctuations as direct indicators of tumor cell death. By exploiting [K⁺]_ex_ dyshomeostasis associated with apoptosis and necrosis, CDPN combines a photosensitizer and a potassium‐sensitive fluorophore within mesoporous silica nanoparticles, encapsulated by a K⁺‐selective membrane for enhanced specificity. In vitro and in vivo studies validate that [K⁺]_ex_ dynamics closely correlate with cell death, enabling precise evaluation of PDT efficacy and data‐driven optimization of the DLI. Using a breast cancer model, CDPN‐guided adjustments identify optimized DLI conditions, achieving significantly improved therapeutic outcomes. This study introduces a new paradigm for PDT, establishing a real‐time, adaptable strategy for guiding treatment parameters and advancing precision oncology.

## Graphical abstract

A cell death self‐reporting photodynamic theranostic nanoagent (CDPN) is developed that integrates photodynamic therapy (PDT) with real‐time K⁺ imaging to provide timely feedback on treatment responses. This approach enables the optimization of the drug‐light interval (DLI), a critical factor in PDT, resulting in significantly enhanced antitumor effectiveness compared to using the maximum accumulation time point of photosensitizer as the DLI.

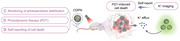

## 1 Introduction

Photodynamic therapy (PDT) is a promising cancer treatment modality that employs light‐activated reactions to generate reactive oxygen species (ROS), inducing selective cell death with the aid of exogenous photosensitizers (PSs).^[^ ^1^, ^2^, ^3^, ^4^ ^]^ PDT offers significant advantages, including high spatial selectivity, minimally invasive nature, and negligible drug resistance, making it an attractive therapeutic option.^[^ ^5^, ^6^, ^7^ ^]^ Conventional monitoring of PDT efficacy typically utilizes the fluorescent signals of PSs to visualize changes in tumor size and assess therapeutic outcomes;^[^ ^8^, ^9^ ^]^ however, this method often encounters delays in treatment feedback,^[^ ^10^, ^11^ ^]^ which can hinder timely optimization of the therapeutic parameters. Given that PDT relies on external light sources for activation, the ability to assess therapeutic responses in situ and in real‐time is crucial for the rapid and precise adjustment of treatment parameters, thereby avoiding excessive or ineffective irradiation, maximizing therapeutic outcomes, and minimizing undesired side effects.^[^ ^12^, ^13^, ^14^, ^15^, ^16^, ^17^, ^18^, ^19^ ^]^ A key factor influencing the effectiveness of PDT is the drug‐light interval (DLI), defined as the time between photosensitizer administration and the initiation of light exposure.^[^ ^20^ ^]^ Most studies currently determine the DLI based on the time point of maximum photosensitizer accumulation at the tumor site,^[^ ^21^, ^22^, ^23^ ^]^ which often overlooks the complex distribution of PSs within the tumor and the subsequent variation in PDT mechanisms across different regions of the tumor,^[^ ^24^ ^]^ potentially failing to identify the optimal DLI. Given that the primary objective of PDT is to induce cell death,^[^ ^25^ ^]^ monitoring the extent of cell death is highly promising for predicting PDT outcomes and optimizing treatment parameters, especially the DLI. However, this remains challenging due to the lack of rationally designed theranostic agents capable of simultaneously performing PDT and self‐reporting cell death in real time.

The dysregulation of cellular cationic gradients, particularly the loss of intracellular potassium (K⁺), is a hallmark of cell death associated with apoptosis and necrosis.^[^ ^26^, ^27^, ^28^, ^29^, ^30^ ^]^ The efflux of K⁺ ions from the cytoplasm to the extracellular space is crucial for initiating and advancing cell death processes.^[^ ^31^, ^32^, ^33^ ^]^ This increase in extracellular K⁺ concentration ([K⁺]_ex_) directly correlates with the extent of cell death, making K⁺ a promising biomarker for evaluating tumor responses to PDT. As such, the development of a K⁺‐sensitive PDT theranostic agent capable of monitoring real‐time changes in [K⁺]_ex_ has the potential to serve as a powerful tool for in situ self‐reporting of cell death, enabling the timely evaluation of PDT efficacy and the optimization of the DLI.

**Figure 1:**
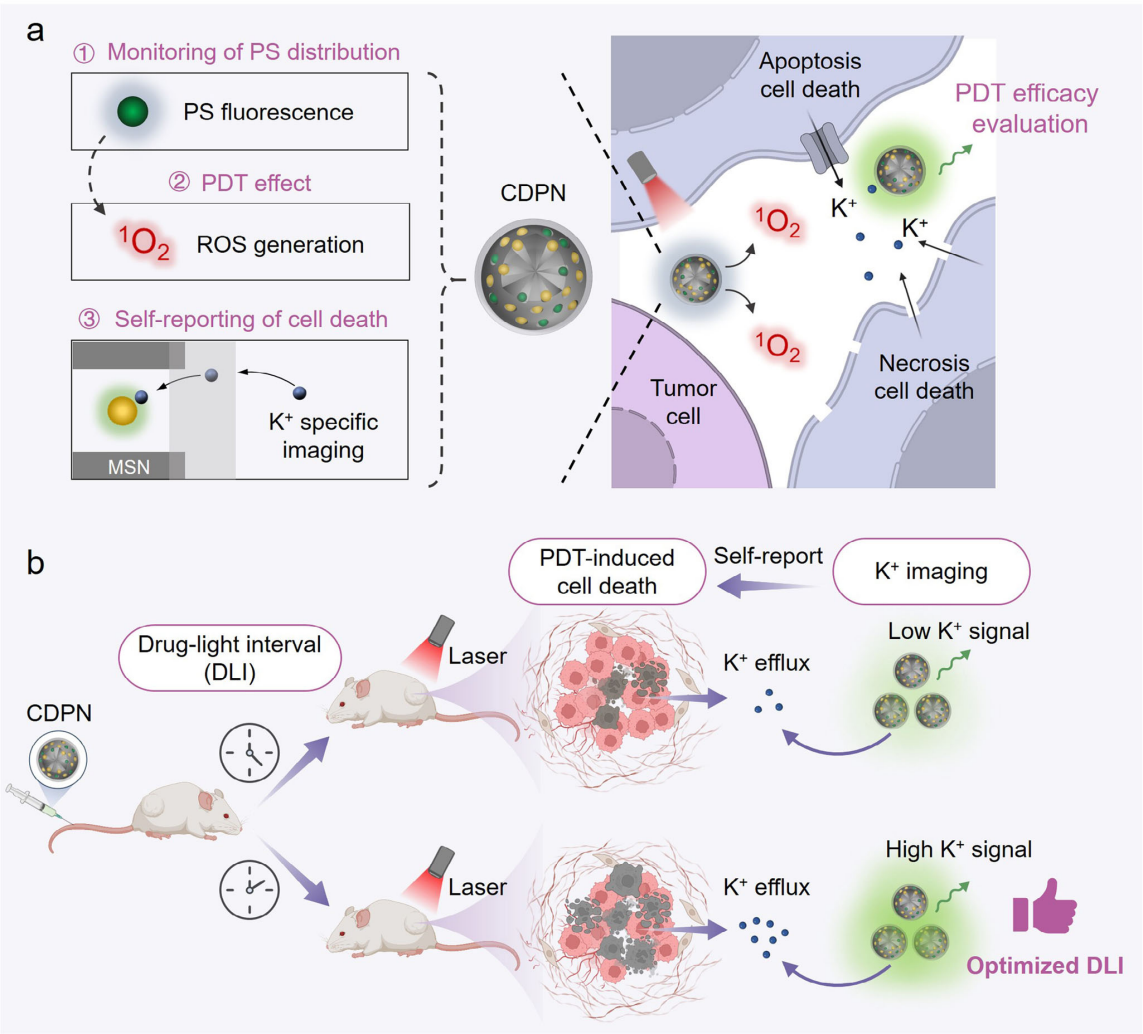
Designing cell death self‐reporting theranostic nanoagents (CDPNs) facilitates real‐time monitoring of tumor responses and enables dynamic optimization of therapeutic strategies. a) CDPNs combine photodynamic therapy (PDT) effects with visualization of photosensitizer (PS) accumulation and self‐reporting of cell death. The reactive oxygen species (ROS) generated by CDPNs trigger cell death, resulting in an increase in extracellular K^+^ concentration. In situ, real‐time monitoring of cell death through K^+^‐specific fluorescence imaging allows for timely evaluation of PDT efficacy. b) As a proof of concept, CDPNs are used to provide prompt feedback on PDT responses and guide the optimization of the drug‐light interval (DLI), a key factor influencing PDT effectiveness, resulting in significantly improved antitumor efficacy.

In this study, inspired by the K⁺ dyshomeostasis associated with cell death, we developed a cell death self‐reporting photodynamic theranostic nanoagent (CDPN) that provides real‐time feedback on PDT responses by monitoring extracellular K⁺ levels (**Figure** **1**). CDPNs integrate the PDT effect and real‐time K⁺ imaging by incorporating a commercially available K⁺ indicator and the photosensitizer chlorin e6 (Ce6) into the hollow cavities of mesoporous silica nanoparticles (MSNs), which are then wrapped with a K⁺‐selective membrane to enhance the specificity and sensitivity of K⁺ imaging. Both in vitro and in vivo experiments demonstrate a positive correlation between increased [K^+^]_ex_ and the degree of PDT‐induced cell death. Furthermore, we successfully employed CDPNs in a breast cancer model to predict PDT outcomes, effectively guiding the optimization of the DLI for significantly enhanced therapeutic efficacy. Our findings underscore the potential of the cell death self‐reporting strategy as a compelling method for the timely and accurate evaluation of PDT efficacy, offering a new approach for guiding treatment plan adjustments and improving patient prognosis.

## 2 Results and Discussion

### 2.1 Design, Fabrication, and Characterization of CDPNs

**Figure 2:**
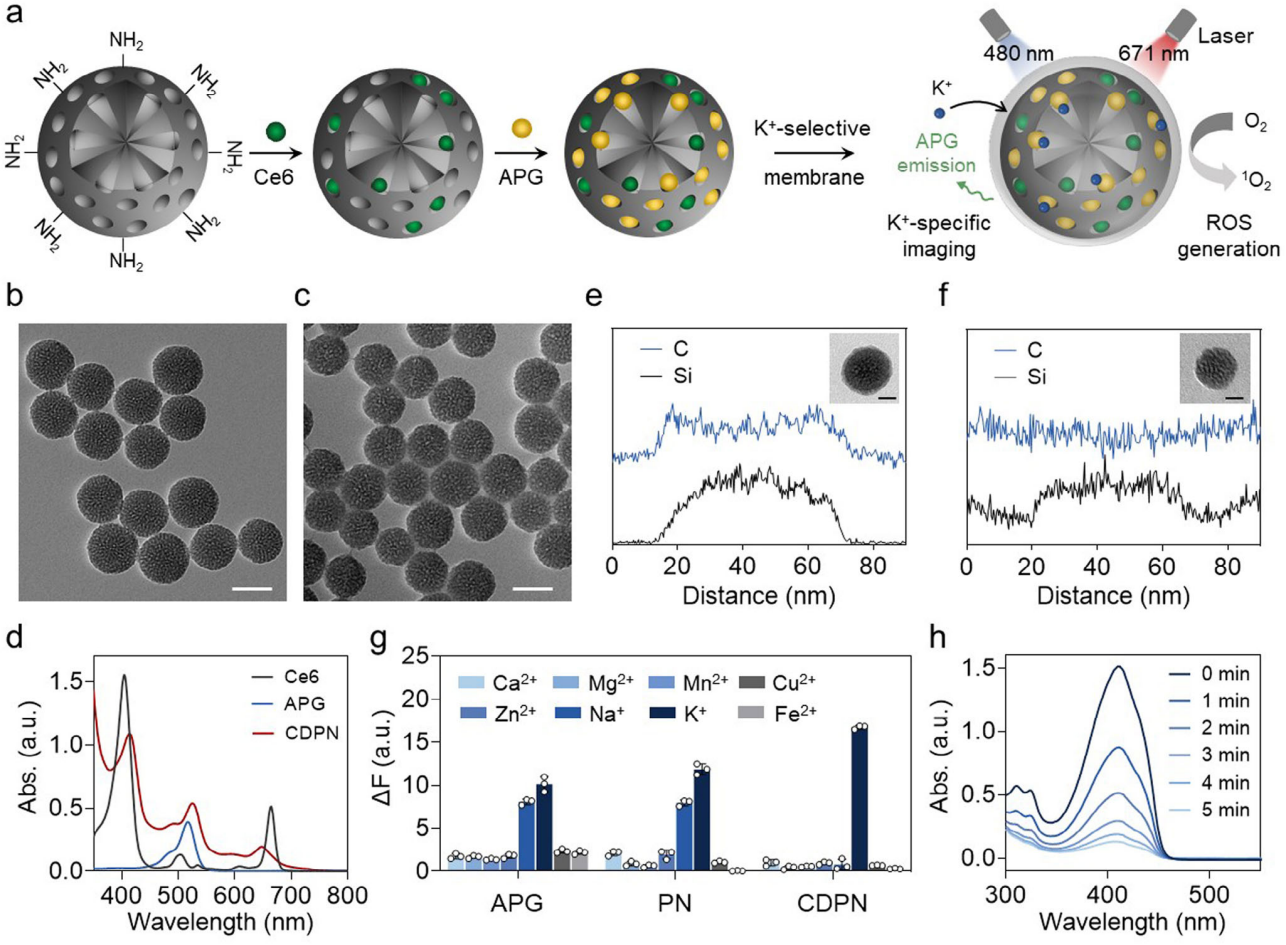
Design and characterization of CDPNs. a) Schematic illustration of the design and synthesis route of the CDPN. TEM images of Ce6‐ and APG‐loaded MSNs b) before and c) after coating with a K^+^‐selective membrane (scale bar = 50 nm). d) UV–vis spectra of APG and CDPNs in DI water, and Ce6 in dimethylsulfoxide. EDS elemental line profiles of e) the CDPN and f) the Ce6‐ and APG‐loaded MSN without a K^+^‐selective membrane coating (PN) and the corresponding high‐resolution TEM images (scale bar = 20 nm). g) Selectivity of APG, PN, and CDPNs toward K^+^ against other physiological cations (Na^+^ 150 mm, K^+^ 150 mm, Ca^2+^ 2 mm, Mg^2+^ 2 mm, Zn^2+^ 2 mm, Mn^2+^ 50 µm, Cu^2+^ 50 µm, Fe^2+^ 50 µm). ΔF is defined as the difference between the fluorescence intensity (F) at a specific ion concentration and the baseline fluorescence intensity (F_0_) in the absence of cation additions. Data are presented as mean ± SD (n = 3). h) Changes in the absorbance spectra of the DPBF probe upon exposure to CDPNs at different irradiation times.

The fabrication procedure of the CDPN is depicted in **Figure** **2**a. In brief, MSNs are surface‐modified with amino groups through a reaction with 3‐aminopropyltriethoxysilane (APTES), resulting in highly uniform nanoparticles with a diameter of ≈65 nm (Figure S1a, Supporting Information). Subsequently, Ce6 and the K^+^ indicator (Asante Potassium Green‐2 tetramethylammonium (TMA^+^) salt, APG) are loaded into the mesopores of MSNs, respectively (Figure 2b). Finally, a K^+^‐selective membrane is constructed on the MSN surface using 3D tripodal ligands (1,1,1‐tris{[(2′‐benzyl‐aminoformyl)phenoxy]methyl}ethane) to improve the sensitivity and selectivity of CDPNs toward K^+^ (Figure 2c; Figure S2, Supporting Information).^[^ ^34^, ^35^ ^]^ Successful loading of Ce6 and APG into MSNs is confirmed by the presence of characteristic absorption peaks in the UV–vis spectra of the nanoparticles (Figure 2d). The UV–vis spectrum reveals a distinct absorption peak at ≈650 nm for Ce6 in dimethylsulfoxide. In contrast, this peak is barely detectable for Ce6 in deionized (DI) water due to its poor solubility in aqueous media (Figure S3, Supporting Information),^[^ ^36^ ^]^ limiting the generation of ROS.^[^ ^37^ ^]^ Notably, the UV–vis spectrum of CDPNs in DI water displays this characteristic peak (Figure 2d), indicating significantly improved water dispersibility of Ce6 when incorporated into MSNs. After modification with the K^+^‐selective membrane, the CDPNs show a hydrodynamic size of ≈106 nm (Figure S1b, Supporting Information), rendering them suitable for drug delivery in vivo.^[^ ^38^ ^]^ Energy‐dispersive X‐ray spectroscopy (EDS) elemental line scanning reveals a carbon element signal, derived from the 3D ligands, exclusively in CDPNs but not in the Ce6‐ and APG‐loaded MSNs without K^+^‐selective membrane coating (Figure 2e,f). These results confirm the successful assembly of the K^+^‐selective 3D ligands on the surface of CDPNs, as supported by Fourier‐transform infrared spectra (Figure S4, Supporting Information).

We next evaluated the K^+^‐sensitive imaging performance and singlet oxygen (^1^O_2_) generation capability of CDPNs. The fluorescence signal of CDPNs is sensitive only to K^+^ (Figure 2g), with its intensity gradually increasing in response to rising K^+^ concentration ([K^+^]) (Figure S5, Supporting Information). In contrast, free APG or Ce6‐ and APG‐loaded MSNs without the K^+^‐selective membrane coating exhibit poor K^+^ selectivity, as their fluorescence signals respond to both K^+^ and sodium ions (Na^+^) (Figure 2g). The superior K^+^ selectivity of CDPNs is attributed to the K^+^‐selective membrane coating, which permits the exclusive passage of K^+^ while blocking other cations. Considering the crucial role of ^1^O_2_ in PDT‐induced cancer cell eradication, we further evaluated the ^1^O_2_ generation capability of CDPNs using 1, 3‐diphenylisobenzofuran (DPBF). The structure of DPBF is destroyed upon interaction with ^1^O_2_, resulting in a decrease in the UV–vis absorption peak at ≈410 nm.^[^ ^39^ ^]^ In the presence of CDPNs, DPBF absorption at ≈410 nm progressively decreases with irradiation time (671 nm laser), indicating rapid ^1^O_2_ generation upon laser exposure (Figure 2h).

### 2.2 CDPN‐Based Extracellular K^+^ Imaging Reflecting the Extent of PDT‐Induced Cell Death

**Figure 3:**
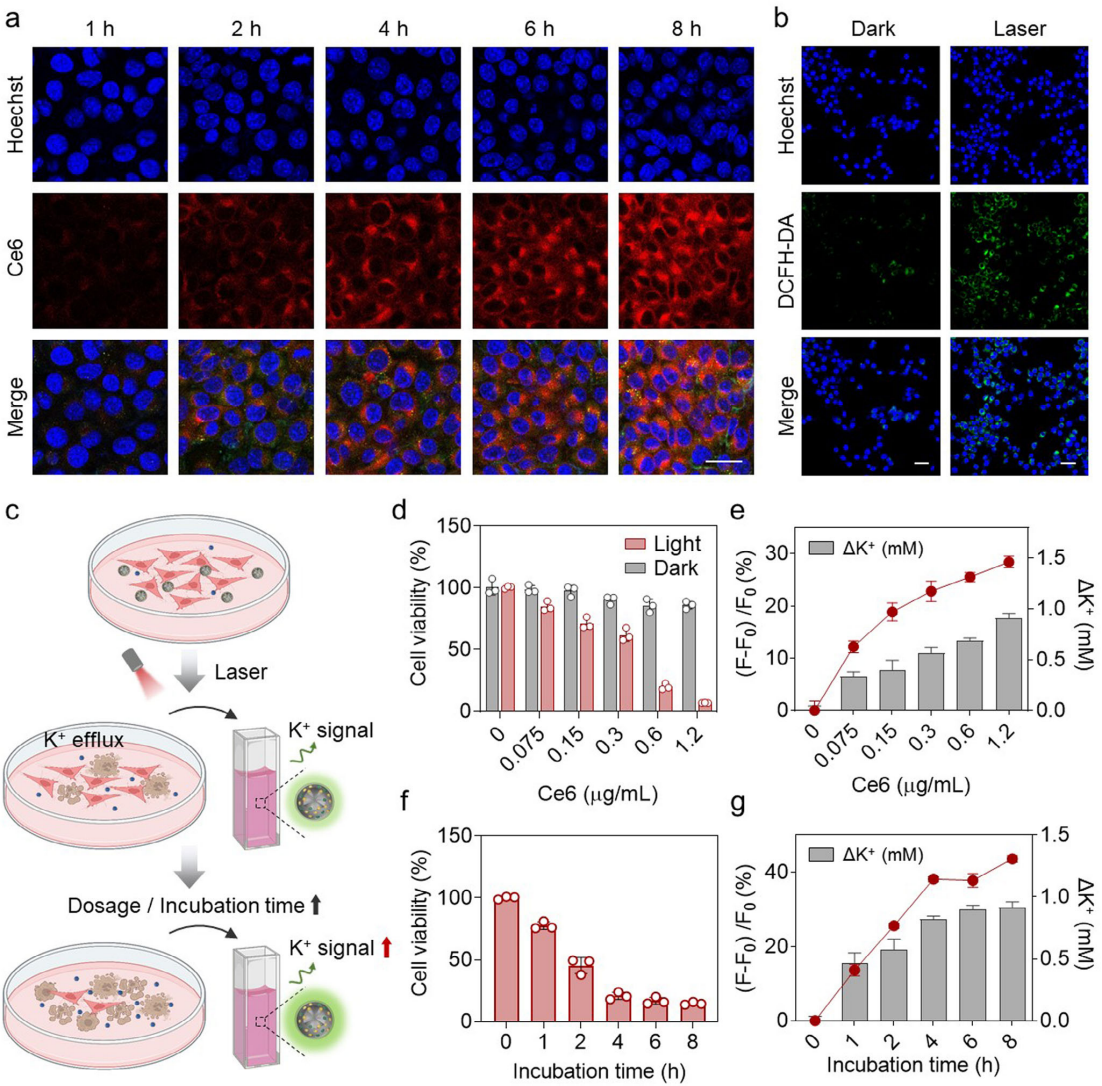
CDPN‐based PDT and extracellular K^+^ imaging for monitoring tumor cell death. a) Time‐dependent confocal laser scanning microscopy (CLSM) of 4T1 cells treated with CDPNs (blue: nucleus; red: Ce6; green: APG) (scale bar = 25 µm). b) CLSM images of intracellular ROS level in 4T1 cells treated with CDPNs (blue: nucleus; green: DCFH‐DA) (scale bar = 50 µm). c) Schematic illustration of CDPN‐based extracellular K^+^ fluorescence imaging for indicating cell death under different PDT conditions. d) Cell viability of 4T1 cells incubated with different concentrations of CDPNs with or without 671 nm irradiation. Data are presented as mean ± SD (n = 3). e) Increase in extracellular K^+^ fluorescence signals (red line) after PDT treatment with varying CDPN concentrations and the corresponding [K^+^] quantification using ICP‐OES (histogram). Data are presented as mean ± SD (n = 3). f) Cell viability of 4T1 cells under different incubation times with CDPNs ([Ce6] = 0.6 µg mL^−1^) with 671 nm irradiation. Data are presented as mean ± SD (n = 3). g) Increase in extracellular K^+^ fluorescence signals (red line) after PDT treatment with varying CDPN incubation times ([Ce6] = 0.6 µg mL^−1^) and the corresponding [K^+^] quantification using ICP‐OES (histogram). Data are presented as mean ± SD (n = 3).

To validate the feasibility of employing CDPNs for assessing PDT efficacy, we investigated the relationship between [K⁺]_ex_ fluctuations and the extent of PDT‐induced cell death using 4T1 cells. Confocal microscopy imaging was performed to investigate the cellular uptake of CDPNs. Fluorescence signals from CDPNs in 4T1 cells are minimal during the first hour of incubation but significantly increase thereafter (**Figure** **3**a; Figure S6, Supporting Information). Intracellular ROS production was monitored using the 2′,7′‐dichlorofluorescein diacetate (DCFH‐DA) probe, which would emit fluorescence upon oxidation by ROS. As shown in Figure 3b, there is a significant increase in the fluorescence intensity of DCFH‐DA after treatment with CDPNs under 671 nm laser irradiation, in stark contrast to the control group without laser exposure, suggesting CDPNs induce intracellular ROS generation and have potential applications in PDT. We further conducted CDPN‐based extracellular K^+^ fluorescence imaging under various PDT conditions, including differing dosages and incubation times (Figure 3c). CDPNs exhibit negligible cytotoxicity against 4T1 cells in the absence of a laser, with cell viability exceeding 85% after 24 h of incubation (Figure 3d). After laser irradiation, CDPNs display a dose‐dependent inhibitory effect on 4T1 cells, reducing relative cell viability to ≈6.4% at a Ce6 concentration of 1.2 µg mL^−1^ (Figure 3d). Moreover, K^+^ fluorescence signals in the culture medium, induced by CDPNs, gradually increase with higher doses (Figure 3e), as confirmed by [K^+^] quantification using inductively coupled plasma optical emission spectrometer (ICP‐OES) (Figure 3e), indicating a rise in [K^+^]_ex_ corresponding to the extent of cell death. Further studies revealed that with prolonged CDPN incubation times, cell death initially increased gradually and then plateaued (Figure 3f), likely due to variations in cellular uptake, consistent with the changes in fluorescence signals and [K^+^] of the culture medium (Figure 3g). These results demonstrate that PDT‐induced cell death is accompanied by K^+^ efflux and an increase in [K^+^]_ex_, which can be effectively detected by CDPNs to evaluate the PDT effect.

### 2.3 Real‐Time In Situ Evaluation of the Therapeutic Responses of PDT Using CDPN

**Figure 4:**
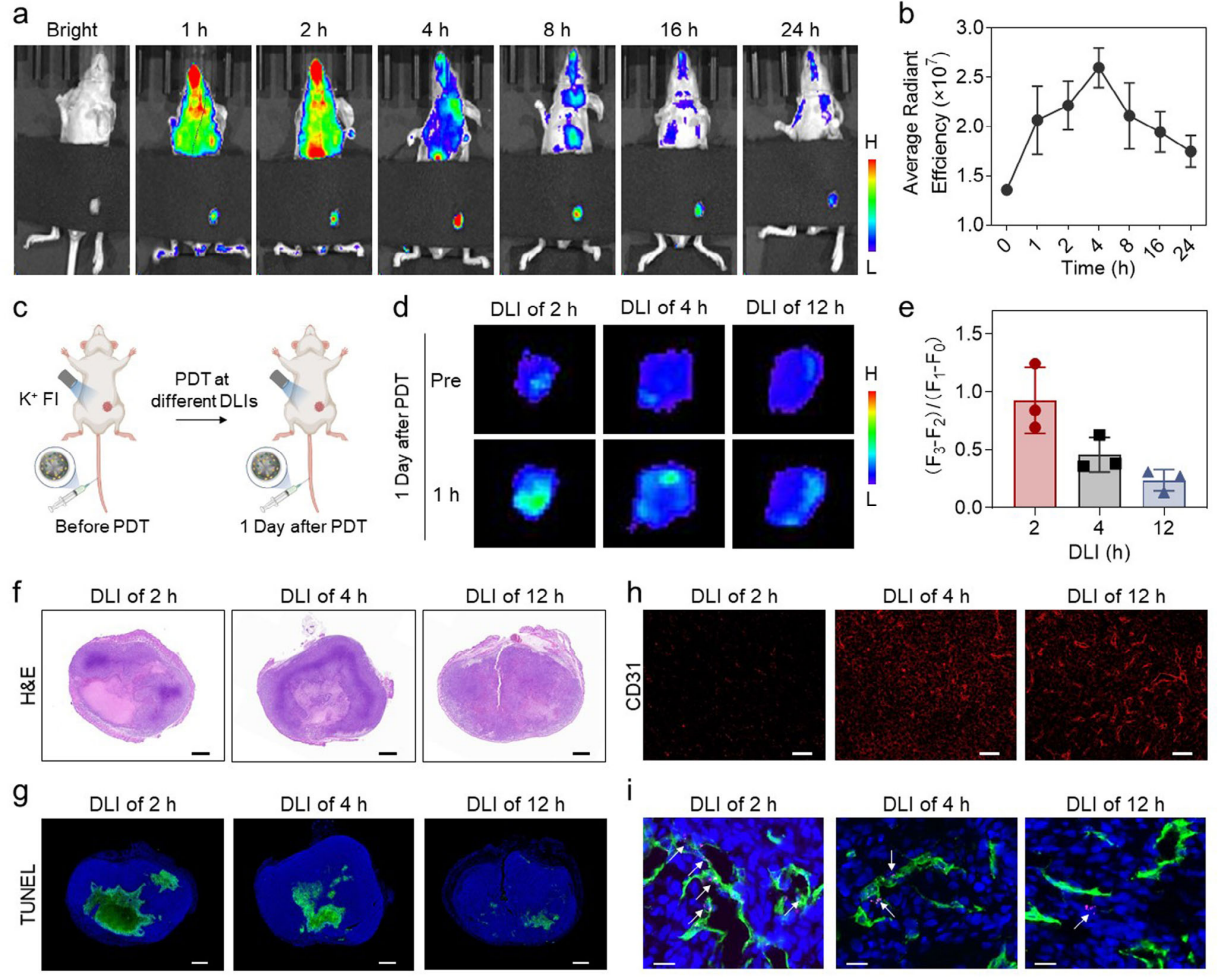
In situ and real‐time evaluation of PDT therapeutic responses using CDPNs. a) Fluorescence imaging of 4T1 tumor‐bearing mice using fluorescence emission from Ce6 at various time points following intravenous (i.v.) injection of CDPNs. b) The average fluorescence intensity at tumor sites over various time points. Data are presented as mean ± SEM (n = 3). c) Schematic illustration of CDPN‐based K^+^ fluorescence imaging (FI) before and after PDT under different DLIs. d) K^+^ imaging of the tumor one day after PDT under different DLIs. e) Quantitative evaluation of changes in fluorescence intensity after PDT under different DLIs. F_0_ and F_1_ represent the fluorescence intensity before and 1 h after the administration of CDPNs prior to PDT. F_2_ and F_3_ represent the fluorescence intensity before and 1 h after the administration of CDPNs following PDT. Data are presented as mean ± SD (n = 3). f) H&E staining (scale bar = 1 mm), g) TUNEL immunofluorescence staining (scale bar = 1 mm), and h) CD31 immunofluorescence staining (scale bar = 50 µm) of the tumor one day after PDT under different DLIs. i) Representative immunofluorescence images of the tumor treated with CDPNs for different times stained with DAPI (blue), CD31 (green) and CDPN‐Cy5 (red) (scale bar = 20 µm).

The cell death self‐reporting capability of CDPNs prompted us to leverage this strategy for evaluating PDT efficacy in vivo and to optimize the DLI. CDPNs were intravenously (i.v.) injected into 4T1 tumor‐bearing mice, and fluorescence imaging revealed a time‐dependent accumulation of CDPNs in the tumor (**Figure** **4**a,b). The fluorescence intensity at the tumor site increases rapidly within 1–4 h post‐injection, reaching a peak at 4 h, indicating gradual accumulation of CDPNs in the tumor. This intensity then gradually declined after 16 h. Furthermore, we randomly divided the breast cancer‐bearing mice into three groups, each receiving PDT at a different DLI of 2, 4, or 12 h, respectively. K^+^ fluorescence imaging of the tumor was performed before and 24 h after PDT to compare changes in K^+^ signals and evaluate treatment efficacy (Figure 4c). As shown in Figure 4d,e, the 2‐h DLI treated group exhibits a stronger fluorescence signal enhancement following PDT compared to the 4‐h and 12‐h DLI groups. This indicates that the 2‐h DLI induces more extensive cell death and superior treatment efficacy among the groups. To validate the accuracy of K^+^ imaging for self‐reporting PDT‐induced cell death, we performed hematoxylin and eosin (H&E) staining and terminal deoxynucleotidyl transferase dUTP nick end labeling (TUNEL) assays on tumor tissues 24 h after treatment. H and E staining shows the most nuclear fragmentation and nucleolysis in the tumors treated with 2‐h DLI (Figure 4f; Figure S7, Supporting Information). Moreover, the green TUNEL signal, indicative of apoptotic cell death, in the 2‐h DLI‐treated mouse is significantly stronger than those in the other groups (Figure 4g). These findings suggest that the maximum accumulation time point of PSs does not necessarily correspond to the optimal DLI, and a 2‐h DLI proves to be more effective for CDPNs in promoting cell death than 4‐h or 12‐h DLIs, aligning with K⁺ imaging results. Additionally, immunofluorescent staining for CD31‐marked vascular endothelial cells reveals significant overlap with CDPNs and extensive vessel damage in the 2‐h DLI group, contrasting sharply with the groups treated with 4‐h or 12‐h DLIs (Figure 4h,i). The combined damage to both vessels and tumor cells likely accounts for the superior PDT efficacy observed with the 2‐h DLI. These findings demonstrate that CDPNs possess in situ self‐reporting capabilities for cell death conditions, enabling real‐time evaluation of PDT outcomes by monitoring cell death‐associated [K⁺]_ex_ changes.

### 2.4 CDPN‐Guided DLI Optimization for Enhanced Therapeutic Outcomes

**Figure 5:**
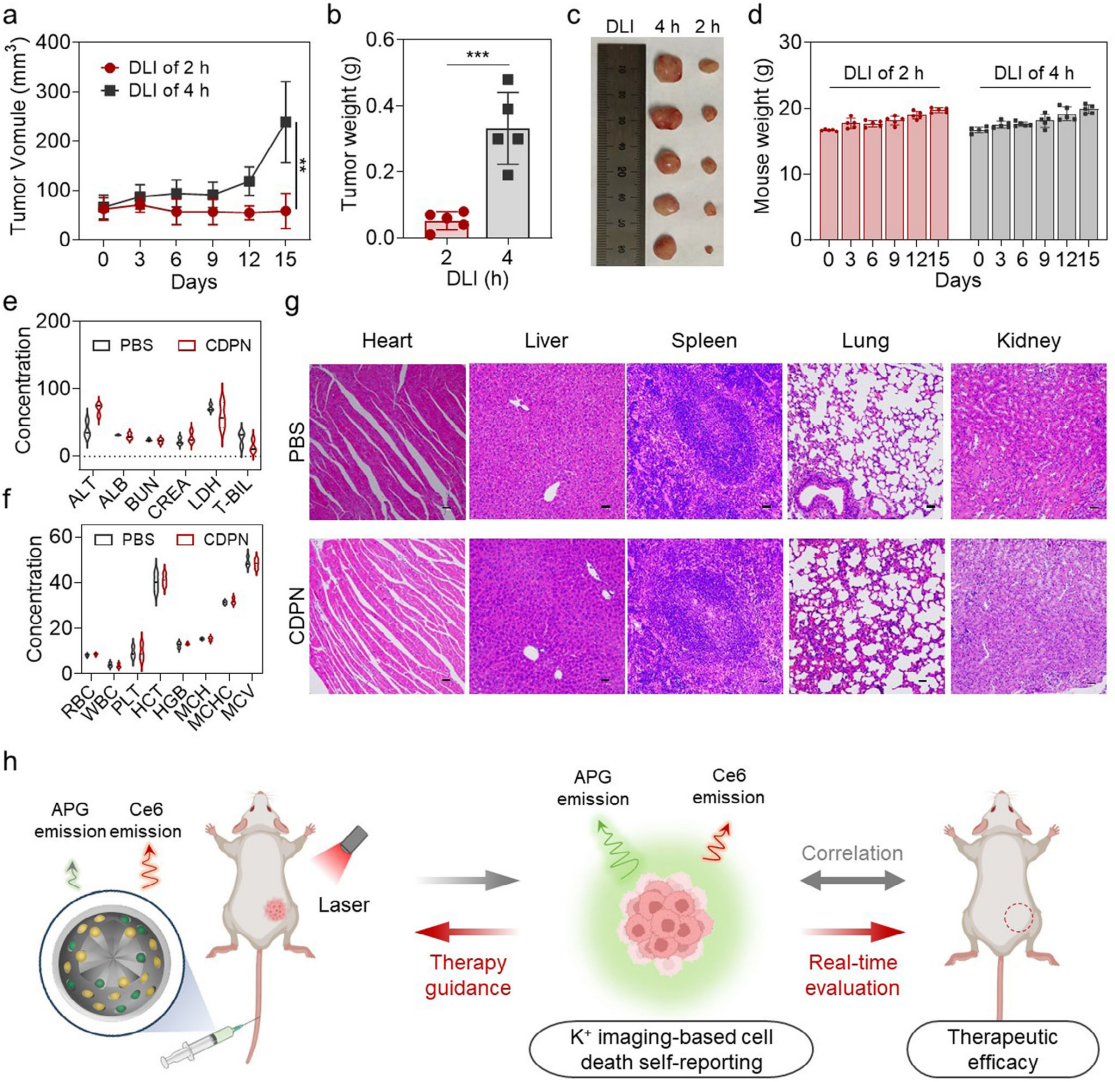
CDPN‐guided DLI optimization for enhanced therapeutic outcomes. a) Tumor growth curves (*p* = 0.0035), b) tumor weight (= 0.0005), and c) tumor growth appearance after PDT with different DLIs at 2 and 4 h. Data are presented as mean ± SD (n = 5). d) Mouse weight during the PDT treatment. Data are presented as mean ± SD (n = 5). e) Biochemical analysis (ALT (U L^−1^): alanine transaminase; ALB (g L^−1^): albumin; BUN (mg dL^−1^): blood urea nitrogen; CREA (µmol L^−1^): creatinine; LDH (10U L^−1^): lactate dehydrogenase; T‐BIL (µmol L^−1^): total bilirubine) and f) hematological parameters (RBC (10^12^ L^−1^): red blood cell; WBC (10^9^ L^−1^): white blood cell; PLT (10^11^ L^−1^): blood platelet; HCT (%): hematocrit; HGB (10g L^−1^): hemoglobin; MCH (pg): mean corpuscular hemoglobin; MCHC (10g L^−1^): mean corpuscular hemoglobin concentration; MCV (fL): mean cell volume) of the mice after treatment with CDPNs or PBS on the 14th day. g) H&E staining of the main organs (heart, liver, spleen, lung and kidney) after i.v. injection of CDPNs or PBS on the 14th day (scale bar = 200 µm). h) Schematic illustration of the CDPN‐based theranostic strategy for real‐time evaluation of therapeutic efficacy and guidance of therapy parameters.

Under the guidance of CDPNs, we further investigated the PDT effects over 15 days using the optimized 2‐h DLI identified via K⁺ imaging and compared it to the 4‐h DLI corresponding to the maximum accumulation time point of CDPNs. Mice treated with a 2‐h DLI show superior inhibition of breast cancer, with significantly smaller tumor sizes compared to those treated with a 4‐h DLI, as observed over a 15‐day period (**Figure** **5**a–c). Throughout the therapy, the body weights of mice increase slowly in all groups (Figure 5d). These results demonstrate the reliability of a CDPN‐based cell death self‐reporting system for evaluating PDT efficacy and guiding the optimization of therapeutic parameters in vivo.

To assess biocompatibility, blood samples from healthy mice were collected for routine blood examinations and biochemical analyses 14 days after intravenous injection of phosphate‐buffered saline (PBS) and CDPNs. The main biochemical indicators and hematological parameters showed no significant differences after CDPN treatment compared to PBS‐treated groups, indicating good blood compatibility, and liver and kidney function safety of CDPNs (Figure 5e,f). Moreover, major organs were extracted 14 days after treatment and then subjected to H&E staining, revealing no apparent morphological changes and tissue damage (Figure 5g). These results demonstrate the biological safety of CDPNs for potential clinical application. Collectively, the CDPN‐based theranostic strategy offers real‐time feedback on PDT responses, facilitating dynamic evaluation and guidance of PDT to enhance antitumor efficacy (Figure 5h).

## 3 Conclusion

In summary, we present a cell death self‐reporting photodynamic theranostic nanoagent (CDPN) that integrates real‐time [K⁺]_ex_ imaging with PDT functionality. By harnessing [K⁺]_ex_ dyshomeostasis as a direct biomarker of cell death, CDPN enables precise, in situ evaluation of therapeutic efficacy and dynamic optimization of the DLI. Our findings, validated in vitro and in vivo using a breast cancer model, demonstrate that the extent of [K⁺]_ex_ changes is a reliable predictor of PDT‐induced cell death. The CDPN‐guided approach identified optimized DLI parameters under specific conditions, significantly improving therapeutic outcomes compared to conventional accumulation‐based methods. This work establishes a transformative paradigm for PDT by shifting from static, accumulation‐driven treatment designs to dynamic, response‐guided therapeutic strategies. Beyond PDT, the cell death self‐reporting strategy may serve as a versatile platform for optimizing treatment regimens in various cancer therapies, providing a pathway toward precision medicine. Future studies could explore the broad applicability of this approach across diverse tumor types and therapeutic modalities, further advancing its clinical translation.

## 4 Experimental Section

### Materials and Reagents

All reagents and solvents were purchased commercially and used without further purification. N‐Hexadecyltrimethylammonium chloride (CTAC), acetonitrile, (3‐aminopropyl)triethoxysilane (APTES), tetraethyl orthosilicate (TEOS), Chlorin e6 (Ce6), and N,N‐dimethylformamide (DMF) were obtained from Aladdin. The commercial K^+^ indicator (APG) was obtained from Abcam. Petroleum ether, anhydrous potassium carbonate, methanol, ethanol, N,N‐dimethylformamide (DMF), sodium chloride (NaCl), and acetonitrile were obtained from Sinopharm Chemical Reagent. 1,3‐Diphenyl isobenzofuran (DPBF) was purchased from MedChemExpress. Roswell Park Memorial Institute (RPMI) 1640 medium and fetal bovine serum (FBS) were purchased from Zhejiang Tianhang Biotechnology Co., Ltd. Penicillin/streptomycin was purchased from Shanghai Titan Technology Co. The reactive oxygen species (ROS) assay kit, enhanced cell counting kit‐8 (CCK‐8), and Hoechst 33 342 staining solutions for live cells were obtained from Beyotime Biotechnology.

### Characterization

Nuclear magnetic resonance (NMR) hydrogen spectrum was acquired using a 400 MHz NMR spectrometer (Bruker, Avance III, Germany). TEM images were obtained with a transmission electron microscope (Thermofisher, Talos L120C G2, USA), while high‐resolution TEM images and the corresponding EDS elemental line profiles were collected using a transmission electron microscope (Thermofisher, TALOS F200X, USA). Dynamic light scattering measurements were performed using a Zetasizer Nano ZS90 instrument (Malvern, UK). Fourier‐transform infrared spectra were obtained with an infrared spectrophotometer (Thermofisher, Nicolet AVA TAR370, USA). UV–vis) spectra were measured with a UV–vis spectrophotometer (Shimadzu, UV‐2600i, Japan). The concentration of K⁺ in samples was quantified using an inductively coupled plasma optical emission spectrometer (PerkinElmer, Avio 500, Singapore).

### Preparation of Ce6‐ and APG‐Loaded MSNs

For the synthesis of MSN, CTAC (1 g) and triethanolamine (0.12 g) were dissolved in 40 mL of DI water and heated to 95 °C. After stabilizing for 1 h, 3 mL of TEOS was added to the solution. The mixture was then stirred at 95 °C for another 1 h. After stopping the reaction, the nanoparticles were centrifuged at 11000 rpm for 30 min and washed with ethanol three times. To remove excess CTAC, the nanoparticles were dialyzed in a 1 wt.% NaCl solution and stirred overnight at 60 °C to obtain MSNs. To modify the surface of MSN with amino groups (MSN‐NH_2_), 20 mg of MSNs were dispersed in 30 mL of ethanol, and 1 mL of APTES was added and dispersed using ultrasound for 3 min. The solution was then stirred at 65 °C for 4 h. After washing with DMF, the nanoparticles were dispersed in 3 mL of DMF. 5 mg of Ce6 dissolved in a minimal volume of DMF was then added to the MSN‐NH_2_ suspension, followed by further dispersion via ultrasound. The mixture was stirred in the dark for 24 h at room temperature. Subsequently, it was centrifuged at 11000 rpm for 20 min and washed three times with DI water, then dispersed in DI water. The Ce6‐loaded MSN solution was mixed with 50 µL of APG (1 mg mL^−1^) in DI water and stirred for 24 h at room temperature. After washing with DI water, the Ce6‐ and APG‐loaded MSN was obtained and dispersed in 2 mL of acetonitrile for further use.

### Preparation of the CDPN

K^+^‐selective 3D tripodal ligands were synthesized following previously reported methods.^[^ ^40^ ^]^ Under vigorous agitation, a 2 mL acetonitrile solution containing 20 mg of the K^+^‐selective ligands was slowly added to a 2 mL Ce6‐ and APG‐loaded MSN acetonitrile ultrasonic suspension. The mixture was kept at 50 °C for 30 min and then annealed at room temperature, followed by washing with methanol and DI water via centrifugation, and finally dispersed in DI water.

### In Vitro K^+^ Fluorescence Imaging Performance of the CDPN

K^+^ aqueous solutions of varying concentrations (0, 10, 30, 60, 90, 120, 140, 150 mm) were prepared. The synthesized CDPN was then exposed to these K^+^ solutions, and the resulting fluorescent signal intensity was measured using a fluorescence spectrophotometer (Cary Eclipse, Agilent Technologies, USA). To account for potential interference from other cations in the living systems, aqueous solutions of common cations were prepared using their respective salts. The free APG, PN, and CDPN were then exposed to these cation solutions, and the fluorescence intensity was measured using the fluorescence spectrophotometer.

### In Vitro PDT Effect of the CDPN

The PDT effect of the CDPN was characterized using DPBF. CDPN was mixed with a DPBF solution, and the experiment was conducted in the absence of light. The UV–vis spectra of DPBF were recorded at different time points to evaluate the ROS production capacity of the CDPN under a 671 nm laser irradiation.

### Cell Culture

4T1 murine breast tumor cells were cultured in RPMI 1640 medium supplemented with 10% FBS and 1% penicillin/streptomycin (100 U mL^−1^) at 37 °C in a 5% CO_2_ atmosphere. The cells were utilized for subsequent experiments during the logarithmic growth phase.

### Cellular Uptake

4T1 cells were seeded into confocal plates at a density of ≈10^5^ cells per plate. After incubation for 24 h, a fresh medium containing CDPNs was added, and the cells were further incubated for 1, 2, 4, 6, and 8 h, respectively. The cells were then washed three times with sterile PBS and fixed with 4% paraformaldehyde for 20 min. Following fixation, the residual solution was washed away with PBS. Then, hoechst 33 342 (10 µg mL^−1^) was added to stain the nuclei for 15 min. Finally, cellular uptake was assessed using a confocal laser scanning microscope (CLSM) (Leica, STELLARIS 5, China).

### Cell Viability Assays

The cytotoxicity of the CDPN against 4T1 cells was assessed using a CCK‐8 assay. 4T1 cells were seeded into a 96‐well plate at a density of 5 × 10^3^ cells per well and cultured for 24 h. The CDPN was then added at various concentrations and incubated with cells for 6 h. Afterward, the medium was removed, and the cells were rinsed with sterile PBS. The cells were irradiated with or without a 671 nm laser for 5 min, and incubation was continued for 18 h. Alternatively, CDPN at the same concentration ([Ce6] = 0.6 µg mL^−1^) was added, and the cells were incubated for different times (0, 1, 2, 4, 6, or 8 h) before being rinsed with sterile PBS. These cells were then irradiated with a 671 nm laser for 5 min, and incubation continued for 16 h. The culture medium containing 10% CCK‐8 was added to each well and incubated at 37 °C for 2 h. Cell viability was assessed by measuring the absorbance at 450 nm using a microplate reader (Agilent, 800TS, USA).

### Intracellular ROS Detection

4T1 cells were seeded in confocal plates at a density of ≈10^5^ cells per plate. After a 24‐h incubation, fresh medium containing CDPN ([Ce6] = 0.6 µg mL^−1^) was added, followed by an additional 6‐h incubation at 37 °C. Following irradiation with a 671‐nm laser at a power density of 1 W for 5 min, the cells were washed three times with sterile PBS and fixed with 4% paraformaldehyde for 15 min. After fixation, the residual fixing solution was washed away with PBS. DCFH‐DA for ROS detection (20 µm) was subsequently added, and after a 20‐min staining period, the cells were washed with sterile PBS. Hoechst 33 342 (10 µg mL^−1^) was then added, and after another 20 min of staining, the cells were washed with sterile PBS. Finally, CLSM was employed to analyze intracellular ROS levels.

### Tumor Model

Female BALB/c nude mice (4–6 weeks old) were purchased from Shanghai SLAC Laboratory Animal Co. Ltd. Animal experiments were conducted in accordance with the guidelines of the Institutional Animal Care and Use Committee of Shanghai Jiao Tong University (IACUC‐O_A2023087). A 100 µL PBS solution containing ≈1 × 10^6^ 4T1 cells was injected into the mammary fat pad of female BALB/c nude mice to establish the breast tumor model.

### In Vivo Biodistribution Analysis

Each mouse received an intravenous (i.v.) injection of 100 µL of CDPN (Ce6 dose: 2 mg kg^−1^). At predetermined time points, fluorescence imaging of the mice was conducted using a fluorescence imaging system (PerkinElmer, IVIS Spectrum, USA) with an excitation wavelength of 640 nm.

### K⁺‐Specific Fluorescence Imaging After PDT In Vivo

K⁺‐specific imaging was conducted before and 1 h after i.v. injection of the CDPN solution using the fluorescence imaging system. Following CDPN administration at 2, 4, and 12 h, the tumor sites were exposed to a 671 nm laser (1 W, 5 min). Subsequently, K⁺‐specific imaging of the mice was performed again 24 h after laser irradiation.

### In Vivo PDT

PDT treatment was initiated when the tumor volume in nude mice reached ≈60 mm^3^. The treatment groups were as follows: group 1 received a 2‐h DLI, and group 2 received a 4‐h DLI, both with an equivalent Ce6 dose of 2 mg kg^−1^ body weight. Laser irradiation was administered by exposing the tumor to a 671‐nm laser (1 W, 5 min). The PDT protocol was repeated 2 days later, with a total of three treatments.

### Tumor Size Measurement

Tumor sizes were measured with a vernier caliper every three days and calculated using the formula: V = (tumor length) × (tumor width)^2^ / 2. Relative tumor volumes were determined as V/V₀, where V₀ represents the tumor volume at the initiation of treatment.

### Biosafety Analysis

Healthy mice were selected and i.v. injected with either CDPN or PBS. On day 14, all mice were sacrificed after blood samples were collected. The major organs, including the heart, liver, spleen, lungs, and kidneys, were analyzed using the H&E staining kit. Biochemical and hematological analyses were conducted on the collected blood samples.

### Statistical Analysis

Data were presented as mean ± standard deviation (SD) unless otherwise specified. GraphPad Prism (version 9.5.1) was used for statistical analysis. Statistical significance was determined using a student's t‐test for comparing two groups. Significant differences are presented as ^*^ *p* < 0.05, ^**^ *p* < 0.01, ^***^ *p* < 0.001 within each experimental group.

## Conflict of Interest

The authors declare no conflict of interest.

## Supporting information



Supporting Information

## Data Availability

The data that support the findings of this study are available from the corresponding author upon reasonable request.
