# Supplementary material for: A Real‐Time Cell Death Self‐Reporting Theranostic Agent for Dynamic Optimization of Photodynamic Therapy
Source: Adv Sci (Weinh). 2025 Mar 8;12(17):2417678. doi: 10.1002/advs.202417678 (PMC12061277; doi:10.1002/advs.202417678)
Supplement: Supplementary file 1 — Supporting Information [file ADVS-12-2417678-s001.docx]

Supporting Information

**A Real-Time Cell Death Self-Reporting Theranostic Agent for Dynamic Optimization of Photodynamic Therapy**

Wei Bian, Qiyue Wang*, Cui He, Pan Tao, Juanjuan Zheng, Yulu Zhang, Jing Li, Fangyuan Li, Hongyan Jia*, and Daishun Ling*

H. Jia, W. Bian^[+]^, J. Li

Department of Breast Surgery, First Hospital of Shanxi Medical University, Taiyuan 030001, China

E-mail: jiahongyan@sydyy.com

W. Bian^[+]^, C. He^[+]^, Y. Zhang

Key Laboratory of Cellular Physiology at Shanxi Medical University, Ministry of Education, Taiyuan 030000, China

Prof. D. Ling, Q. Wang^[+]^, P. Tao, J. Zheng

Frontiers Science Center for Transformative Molecules, School of Chemistry and Chemical Engineering, School of Biomedical Engineering, National Center for Translational Medicine, Shanghai Jiao Tong University, Shanghai 200240, China
E-mail: [dsling@sjtu.edu.cn](mailto:dsling@sjtu.edu.cn); wangqiyue@sjtu.edu.cn

Prof. F. Li, Q. Wang^[+]^

Department of Clinical Laboratory, Songjiang Research Institute, Shanghai Key Laboratory of Emotions and Affective Disorders (LEAD), Songjiang Hospital Affiliated to Shanghai Jiao Tong University School of Medicine, Shanghai 201600, China

[+] These authors contributed equally to this work.

Keywords: theranostic agent; photodynamic therapy; nanosensor; fluorescence imaging; imaging-guided surgery

**Supplementary Figures**

**
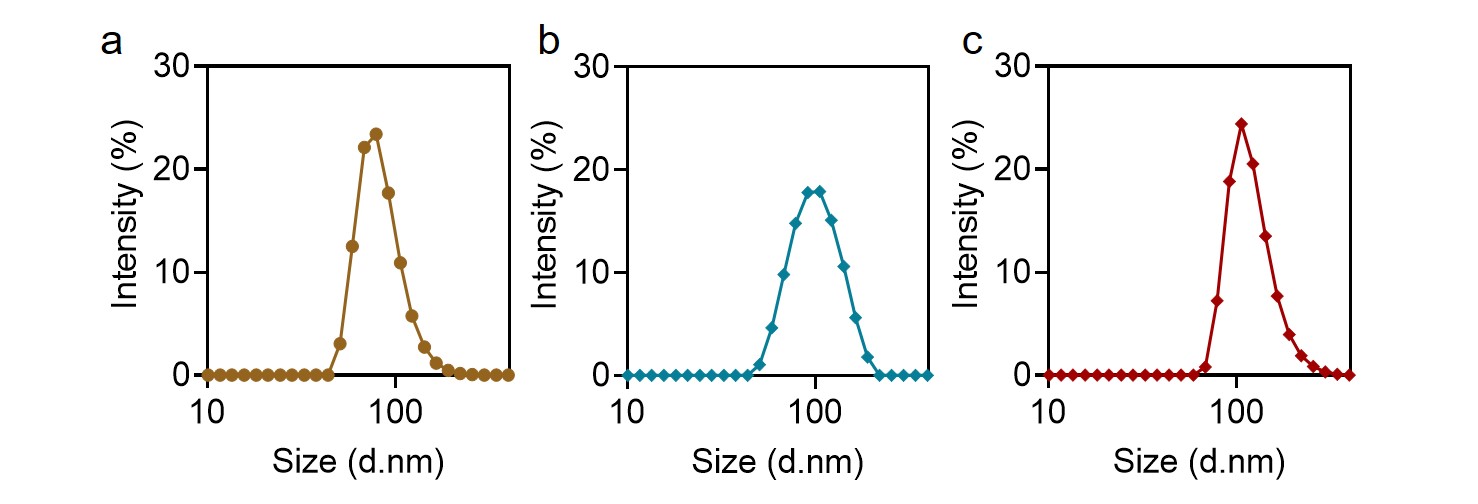
**

**Figure S1.** The hydrodynamic size distributions of (**a**) mesoporous silica nanoparticles (MSNs), (**b**) Ce6-loaded MSNs and (**c**) cell death self-reporting photodynamic theranostic nanoagents (CDPNs).

**
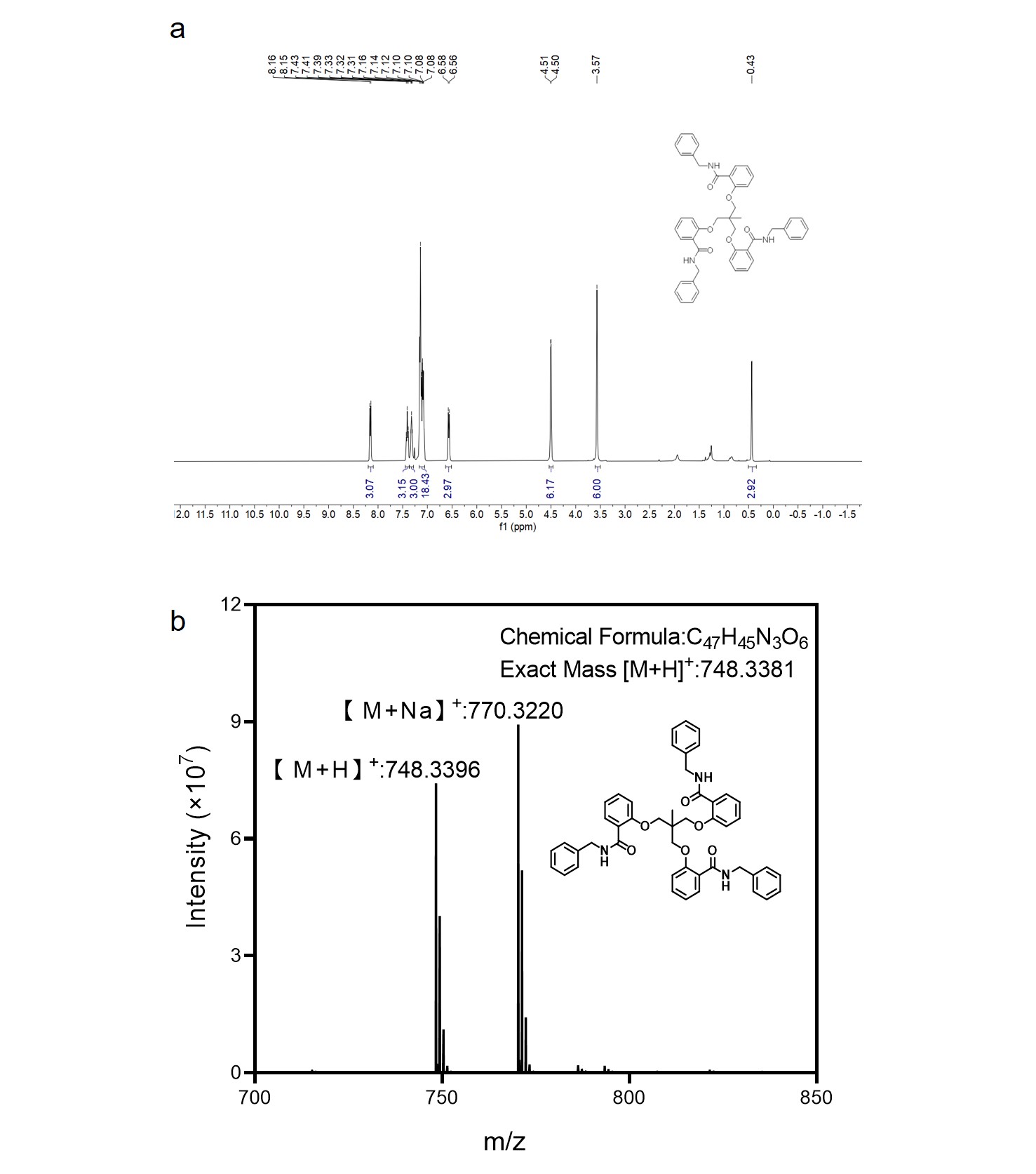
**

**Figure S2. (a)** ^1^H NMR analysis of K^+^-selective 3D ligands. ^1^H NMR (400 MHz, CDCl_3_) δ: 8.15 (d, *J* = 7.7 Hz, 3H, NH), 7.45 - 7.37 (m, 3H, ArH), 7.33 (t, *J* = 5.6 Hz, 3H, ArH), 7.17 - 7.06 (m, 18H, ArH), 6.57 (d, *J* = 8.3 Hz, 3H, ArH), 4.50 (d, *J* = 2.9 Hz, 6H, NHCH_2_), 3.57 (s, 6H, OCH_2_), 0.43 (s, 3H, CH_3_). **(b)** High resolution mass spectrometry (HRMS) (ESI) calcd. for the K^+^-selective ligand, [M+H]^+^ 748.3381, found 748.3396; HRMS (ESI) calcd. for the ligand, [M+Na]^+^ 770.3201, found 770.3220.


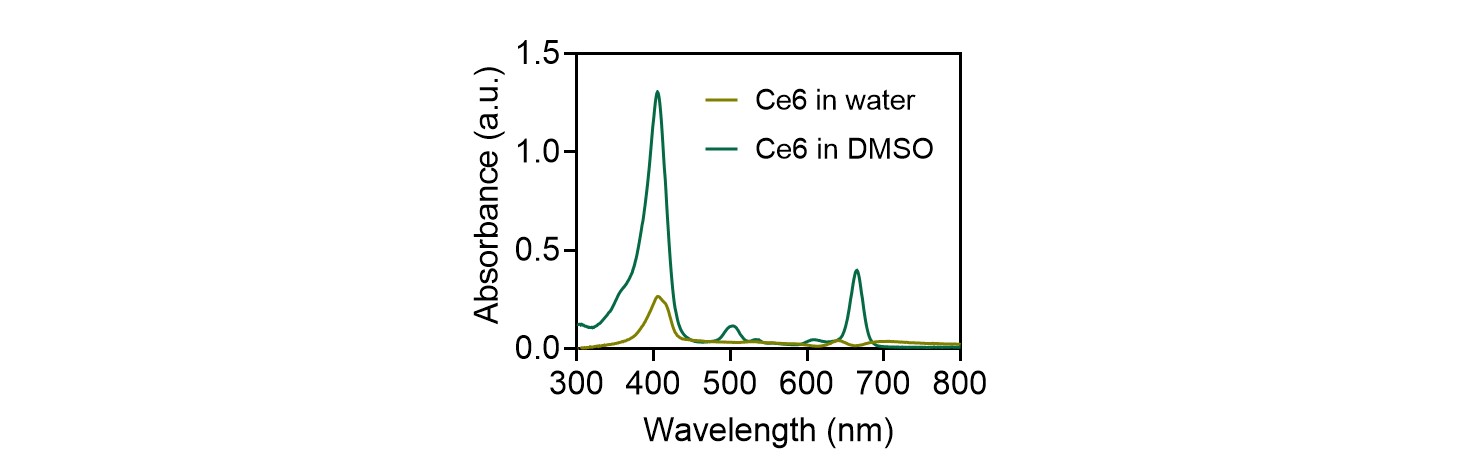


**Figure S3.** Ultraviolet-visible spectra of free Ce6 dispersed in deionized water or in dimethylsulfoxide (DMSO).


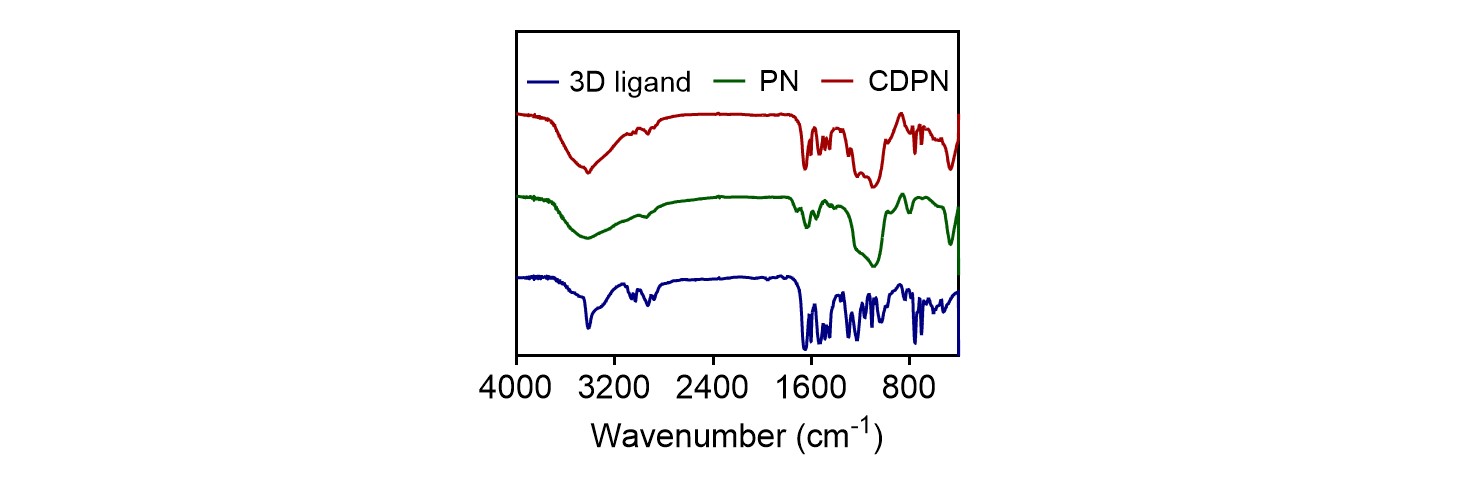


**Figure S4.** Fourier-transform infrared spectra of the K^+^-selective ligand, Ce6 and APG-loaded MSN without a K^+^-selective membrane coating (PN), and CDPN. CDPNs show characteristic bands for the K^+^-selective ligand, indicating successful ligand modification.


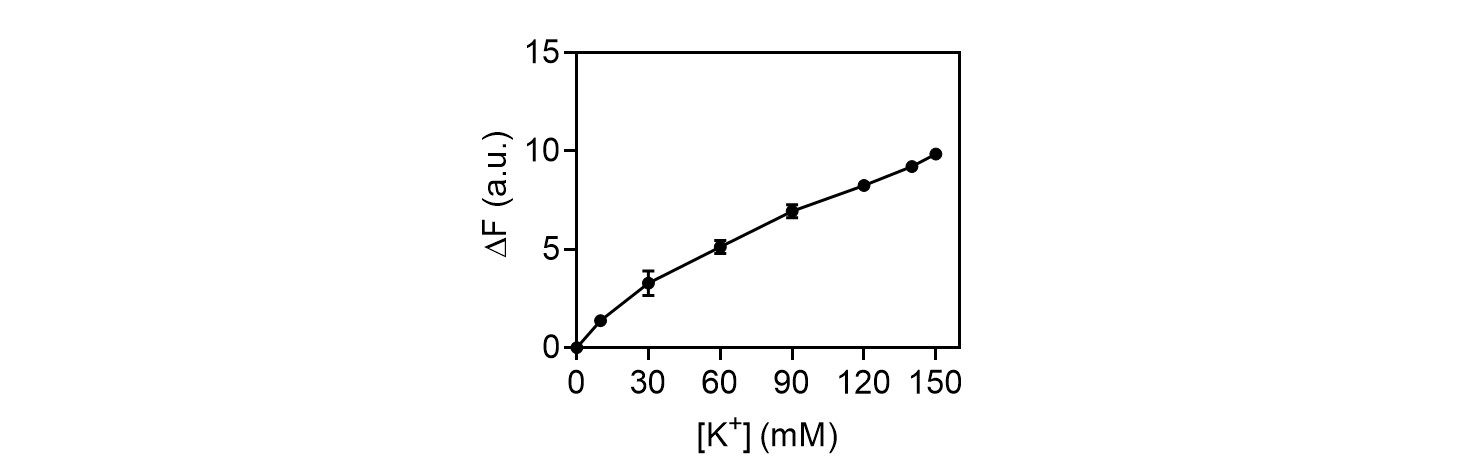


**Figure S5.** Changes in the fluorescence intensity of CDPNs in aqueous solutions with K^+^ concentration ([K^+^]) increasing from 0 to 150 mM. Data are presented as mean ± SD (n = 3).


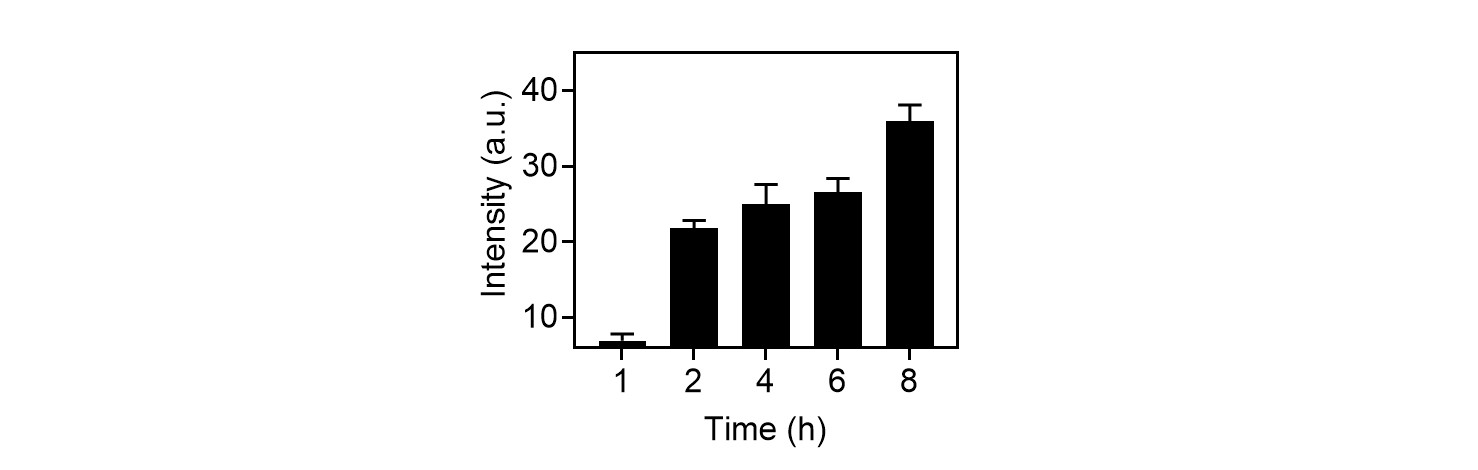


**Figure S6.** The average fluorescence intensity of 4T1 cells treated with CDPNs over various time points. Data are presented as mean ± SD (n = 3).

**
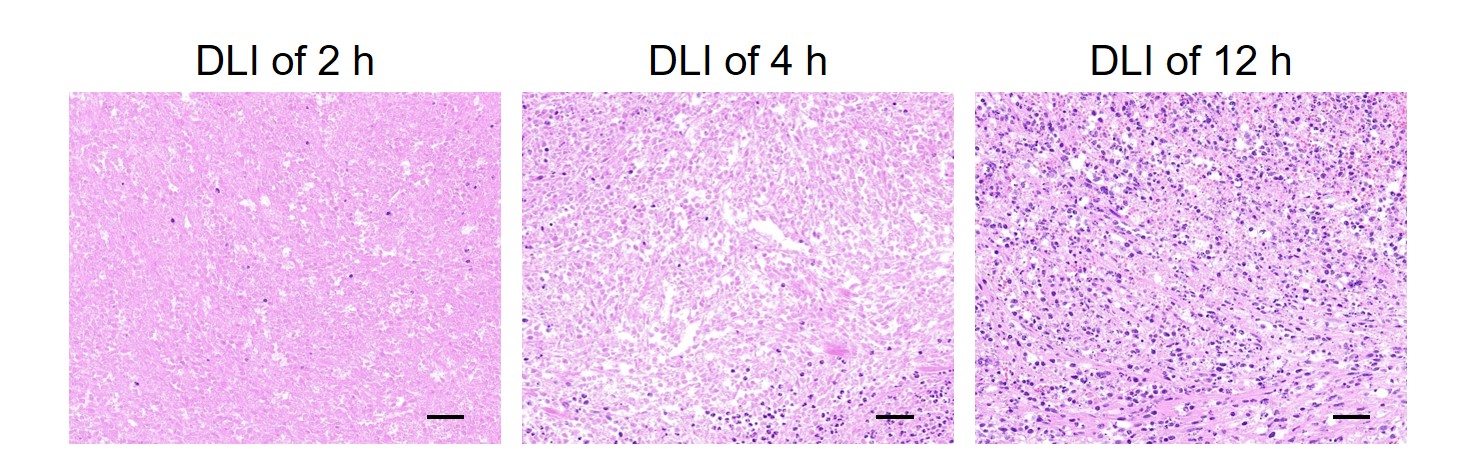
**

**Figure S7.** H&E staining of the tumor one day after PDT under different drug-light intervals (DLIs) (scale bar = 50 μm).
